# Transient Mid-Ventricular Ballooning Syndrome: An Atypical Variant of Stress Cardiomyopathy

**DOI:** 10.7759/cureus.35537

**Published:** 2023-02-27

**Authors:** Andrew Takla, Amir Mahmoud, Mostafa R Mostafa, Deeptanshu Jain

**Affiliations:** 1 Internal Medicine, Rochester Regional Health, Rochester, USA

**Keywords:** takotsubo cardiomyopathy (ttc), takotsubo syndrome, broken heart syndrome, atypical takotsubo, stress-related cardiomyopathy

## Abstract

Takotsubo cardiomyopathy, also known as stress cardiomyopathy (SCM) or broken heart syndrome, is characterized by transient systolic dysfunction of the left ventricle (LV). It typically affects the apical segment, but several rare variants exist. This report represents a rare variant of atypical stress cardiomyopathy that mimics territorial regional wall motion abnormalities of a blocked epicardial vessel.

## Introduction

Takotsubo cardiomyopathy, also known as stress cardiomyopathy (SCM) or broken heart syndrome, was first reported in 1990 and is characterized by transient systolic dysfunction of the left ventricle (LV) [1]. The disorder has been increasingly recognized worldwide with an estimated prevalence of 1%-2% of acute coronary syndrome (ACS) presentations [2]. Multiple diagnostic criteria have been established [3], and several variants have been described in the literature [4]. This report represents a rare variant of atypical SCM that mimics territorial regional wall motion abnormalities of a blocked epicardial vessel.

## Case presentation

This is a 46-year-old female patient with a past medical history of hypertension, hyperlipidemia, Graves’ disease currently in remission, gastroesophageal reflux disease, anxiety, and chronic migraine headaches who presented to the emergency department with exertional retrosternal chest pain that has been occurring intermittently for the past two weeks after the loss of her family member. The pain was associated with dyspnea and occasional palpitation. The patient at baseline had a good exercise tolerance. One day before admission, she started to experience severe intractable continuous chest pain radiating to her back and left shoulder without relief, which prompted her to come to the emergency department. Pertinent home medications include propranolol, citalopram, and chlorthalidone. Family history was negative for sudden cardiac death or ischemic cardiovascular events. Her pertinent social history includes smoking half a pack of cigarettes per day for one year. She also has a history of allergy to aspirin.

The patient was afebrile, vitally stable on admission, and normotensive with a blood pressure of 130/80 mmHg, a heart rate of 68 beats per minute, a respiratory rate of 16 per minute, and a saturation of 99% on room air. The rest of the systemic examination was unremarkable, apart from Graves’ ophthalmopathy.

**Figure 1:**
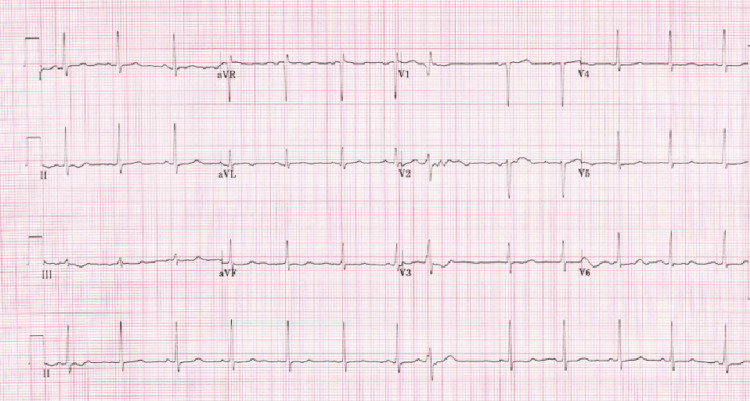
EKG showing 0.5 mv ST depressions in leads II and III and 0.5 mv ST elevation in lead aVL. EKG, electrocardiogram; aVL, augmented vector left; aVR, augmented vector right; avF, augmented vector foot

Laboratory workup revealed normal complete blood count, complete metabolic panel with kidney functions and liver functions at baseline, hemoglobin A1C of 5.2%, low-density lipoprotein of 91 mg/dl, thyroid-stimulating hormone of 1.6 ulU/ml, and elevated high-sensitivity troponin of 572 pg/ml on admission with a negative delta of 89 pg/ml at three hours. Electrocardiogram (Figure 1) was normal sinus with 0.5 mv ST depressions in leads II and III and 0.5 mv ST elevation in lead augmented vector left (aVL).

**Figure 2:**
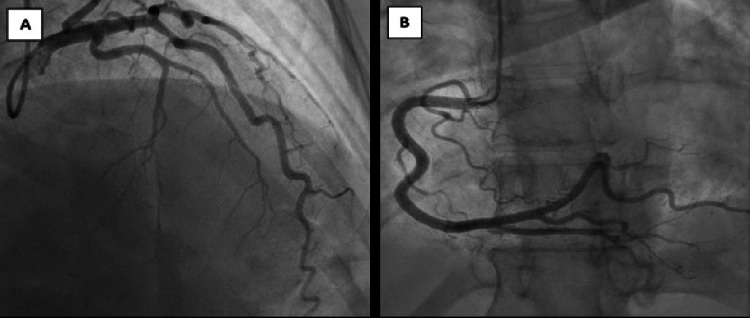
A) Coronary angiogram showing normal left anterior descending and left circumflex. B) Coronary angiogram showing normal right coronary artery.

Transthoracic echo revealed regional wall motion abnormalities in the form of severe hypokinesis in the mid-LV cavity particularly affecting the mid-anterolateral and mid-inferolateral walls with moderately reduced LV systolic function ejection fraction of 40%, mild mitral regurgitation, and mild tricuspid regurgitation. The patient was taken to the cath laboratory after being loaded with dipyridamole, and her coronary angiography (Figure 2) revealed normal coronary anatomy, suggesting that cardiomyopathy is probably stress-induced (mid-wall variant versus focal variant Takotsubo cardiomyopathy).

The patient was followed up by the heart failure team and was discharged after two days on guideline-directed medical therapy with carvedilol 6.25 mg twice daily, sacubitril-valsartan 24-26 mg twice daily, dapagliflozin 10 mg once daily, and spironolactone 25 mg once daily, for further follow-up in the outpatient clinic.

## Discussion

Stress cardiomyopathy (SCM) is characterized by transient wall motion abnormalities that usually extend beyond a single epicardial vessel distribution and a variable degree of ventricular dysfunction that depend on the extent of myocardial stunning. It mimics acute coronary syndrome (ACS) in its presentation: patients usually present with acute substernal chest pain (75.9%), dyspnea (46.9%), or syncope (7.7%), as well as electrocardiographic changes and modest elevation in the cardiac biomarkers, but in the absence of angiographic evidence of obstructive coronary artery disease or acute plaque rupture [1,2].

The disorder is believed to be precipitated by physical stress (~40%), emotional stress (~30%), or acute medical illness, hence its nomenclature [5,6]. The underlying mechanism of myocardial stunning is not well understood but is postulated to be related to excess catecholamine release leading to diffuse microvascular spasm or direct catecholamine-associated myocardial toxicity [7-10]. Some studies have also recognized a familial pattern of the disorder, but no genetic mutation has been identified [11,12]. The disease disproportionately affects females (~90%) and older adults with a mean age of 66.4 years [13]. It commonly (~80%) affects the apex of the left ventricle (LV) causing apical ballooning in an octopus’ trap shape and hence the name “Takotsubo.”

The mid-ventricular ballooning syndrome is an atypical variant of stress cardiomyopathy that is characterized by transient hypokinesis in the mid-segments of the left ventricle with apical sparing [14]. It represents around 14.6% of patients. Other atypical variants include the basal variant (2.2%) affecting basal segments, the focal variant (1.5%) affecting an isolated segment, and rarely a global variant with hypokinesis of all ventricular segments [15,16]. The atypical variants tend to present in younger patients with a lower prevalence of hypertension [17]. The stressor type itself appears to modify the pattern of LV dysfunction, with more frequent surgical and disease-related stressors in patients with the atypical variants [18]. Clinically, unlike the typical variant, these patients usually present with lower brain natriuretic peptide levels, higher ejection fraction, and ST segment depressions rather than elevations [16].

Stress cardiomyopathy has a favorable course and usually recovers completely with a conservative medical approach [19,20]. Complications including acute heart failure, acute valvular dysfunction, dynamic left ventricular outflow tract obstruction, arrhythmias, sudden cardiac arrest, and cardiogenic shock have been reported in up to 20% of cases and predominantly affect males; however, there was no significant difference reported regarding the clinical outcomes between the typical and atypical variants [5]. Very limited data exist on the duration of medical therapy in such patients; however, the expert consensus suggests treatment for up to 12 months [21]. Recurrent cases have been reported in the literature; therefore, it is important to manage the underlying psychiatric illness and treat comorbid anxiety and depression. In a study of 100 patients followed up over around 4.5 years, the recurrence rate of the disorder was reported to be 11.4% [22].

Differential diagnosis

The differential diagnosis of a middle-aged female patient who presented with ACS, newly diagnosed cardiomyopathy, territorial regional wall motion abnormalities, and normal coronary anatomy can be challenging. Thyroid cardiomyopathy in a patient with an underlying history of Graves’ disease should be on top of the differentials. Thyroid hormones have similar effects to catecholamine-mediated sympathetic stimulation of beta-adrenergic receptors [23], and multiple cases have been reported in the literature with Takotsubo cardiomyopathy in the setting of abnormal thyroid hormone levels including atypical variants: mid-wall variant Takotsubo [24]. Graves’ disease in our case was clinically in remission, and thyroid-stimulating hormone was also within normal limits making hyperthyroid cardiomyopathy less likely; however, it is important to highlight the role of thyroid function testing in clinically appropriate settings.

Additionally, the patient’s medication list included citalopram. There is a possible suggested association between serotonin-norepinephrine reuptake inhibitors (SNRI) and selective serotonin reuptake inhibitors (SSRI) and Takotsubo cardiomyopathy; however, a causal relationship has not yet been established. Twenty-one cases with SNRI and six cases with SSRI have been reported to develop Takotsubo cardiomyopathy without a prior history of emotional or physical stress in a study by Woronow et al. [25]. In our case, the patient was started on citalopram six months prior to admission. It was reported that patients on SSRI can develop Takotsubo at doses even below the maximum recommended dose [25]. This could be concerning given that SSRIs are widely used in the United States [26]. Alternatively, SSRI and cognitive behavioral therapy were arguably suggested as a treatment for patients with recurrent stress cardiomyopathy [27]. Further research is needed to shed more light on such an association.

## Conclusions

Stress cardiomyopathy can present with atypical variants that mimic territorial regional wall motion abnormalities of a blocked epicardial vessel and can be very challenging to diagnose. It is unclear at the moment if these atypical variants are of specific clinical significance; however, prompt coronary angiography is warranted in all cases to rule out acute coronary syndrome.
